# The Seroepidemiology of *Haemophilus influenzae* Type B Prior to Introduction of an Immunization Programme in Kathmandu, Nepal

**DOI:** 10.1371/journal.pone.0085055

**Published:** 2014-01-22

**Authors:** Andrew S. J. Marshall, Charlotte I. S. Barker, Anoop S. Pulickal, Elizabeth Kibwana, Samir C. Gautam, Elizabeth A. Clutterbuck, Stephen M. Thorson, Shrijana Shrestha, Neelam Adhikari, Andrew J. Pollard, Dominic F. Kelly

**Affiliations:** 1 Oxford Vaccine Group, Oxford University, and the NIHR Oxford Biomedical Research Centre, Oxford, United Kingdom; 2 Patan Hospital, Kathmandu, Nepal; University of Cambridge, United Kingdom

## Abstract

*Haemophilus influenzae* type b (Hib) is now recognized as an important pathogen in Asia. To evaluate disease susceptibility, and as a marker of Hib transmission before routine immunization was introduced in Kathmandu, 71 participants aged 7 months–77 years were recruited and 15 cord blood samples were collected for analysis of anti-polyribosylribitol phosphate antibody levels by enzyme-linked immunosorbent assay. Only 20% of children under 5 years old had levels considered protective (>0.15 µg/ml), rising to 83% of 15–54 year-olds. Prior to introduction of Hib vaccine in Kathmandu, the majority of young children were susceptible to disease.

## Introduction

*Haemophilus influenzae* type b (Hib) remains a significant cause of invasive bacterial disease globally, and is of particular importance in resource-poor countries. It is estimated to cause approximately 371,000 deaths each year and over 8 million cases of serious disease in children under 5, including pneumonia, meningitis and epiglottitis [1]. The Hib vaccine comprises the Hib surface polysaccharide antigen polyribosylribitol phosphate (PRP) conjugated to a protein carrier. Since 2006, the World Health Organization (WHO) has recommended that this vaccine be included in all routine infant immunization programmes [2]. The WHO advised that “lack of local surveillance data should not delay the introduction of these vaccines”, especially in countries with evidence of a high burden of disease. Despite this, uncertainty has surrounded the relative importance of Hib disease in South East Asia, and adoption of the vaccine has subsequently been slow [3]. With support from the Global Alliance for Vaccines and Immunization, Hib vaccine was introduced in Nepal in 2009 [4].

In unvaccinated populations, repeated exposure to Hib antigens during childhood is thought to lead to natural immunity, with development of protective levels of anti-PRP antibody over time [5]. The aim of this seroepidemiological study, carried out in the pre-Hib-vaccination era in Nepal, was to determine the level of Hib-specific serum antibodies in a sample of the Kathmandu population, in order to evaluate disease susceptibility, and as a surrogate marker of Hib transmission prior to vaccine introduction. In addition these data may provide a useful baseline against which to compare post-vaccination seroepidemiological studies, for example when determining whether a booster vaccine dose will be needed in this population [6].

## Materials and Methods

### Ethics statement

As part of the original study [7], ethical approval was given by the appropriate Institutional Review Boards (The Nepal Health Research Council and the Oxford Tropical Research Ethics Committee, reference 017-05) for sample storage and use in future studies of vaccine-related immunity. Written consent was provided by all participants or the parents/guardians for those under the age of 18 years.

### Participants and samples

The samples were collected as part of a seroepidemiological study described previously [7]. Briefly, patients attending the outpatient department at Patan Hospital, Kathmandu, Nepal, for non-infectious conditions in June and July 2006 were invited to participate. Hib vaccine was available in some private clinics in Nepal at the time, but in practice unavailable to the vast majority of patients attending Patan Hospital. Following fully informed consent from the patient (or guardian if the patient was <18 years old) venous blood samples were taken. Cord blood samples were obtained from consecutive deliveries in the Patan Maternity Ward where consent was provided. Volunteers with pyrexia, or any immune disorder were excluded. Sera were separated from clotted whole-blood samples by centrifugation and frozen.

### Hib ELISA

Serum anti-PRP antibody concentrations were determined using a standard protocol [8], [9]. Duplicate sample sera, initially diluted 1∶20, were re-tested at 1∶200 and/or 1∶2000 if necessary.

### Statistical analysis

The mean concentration for each sample duplicate was log_10_-transformed for calculation of the standard error of the geometric mean concentration (GMC) [10]. Any sample below the lower limit of detection (0.1 µg/ml) was given a value of 0.08 µg/ml for the purpose of analysis. Samples were categorized as cord blood or by age of participant in completed years. There is limited evidence regarding the concentration of anti-PRP antibody required for protection from invasive disease [11], [12]. As in previous studies, we used 0.15 µg/ml and 1 µg/ml of anti-PRP antibody as thresholds for short- and long-term protection respectively [8], [13], [14].

## Results

**Figure 1:**
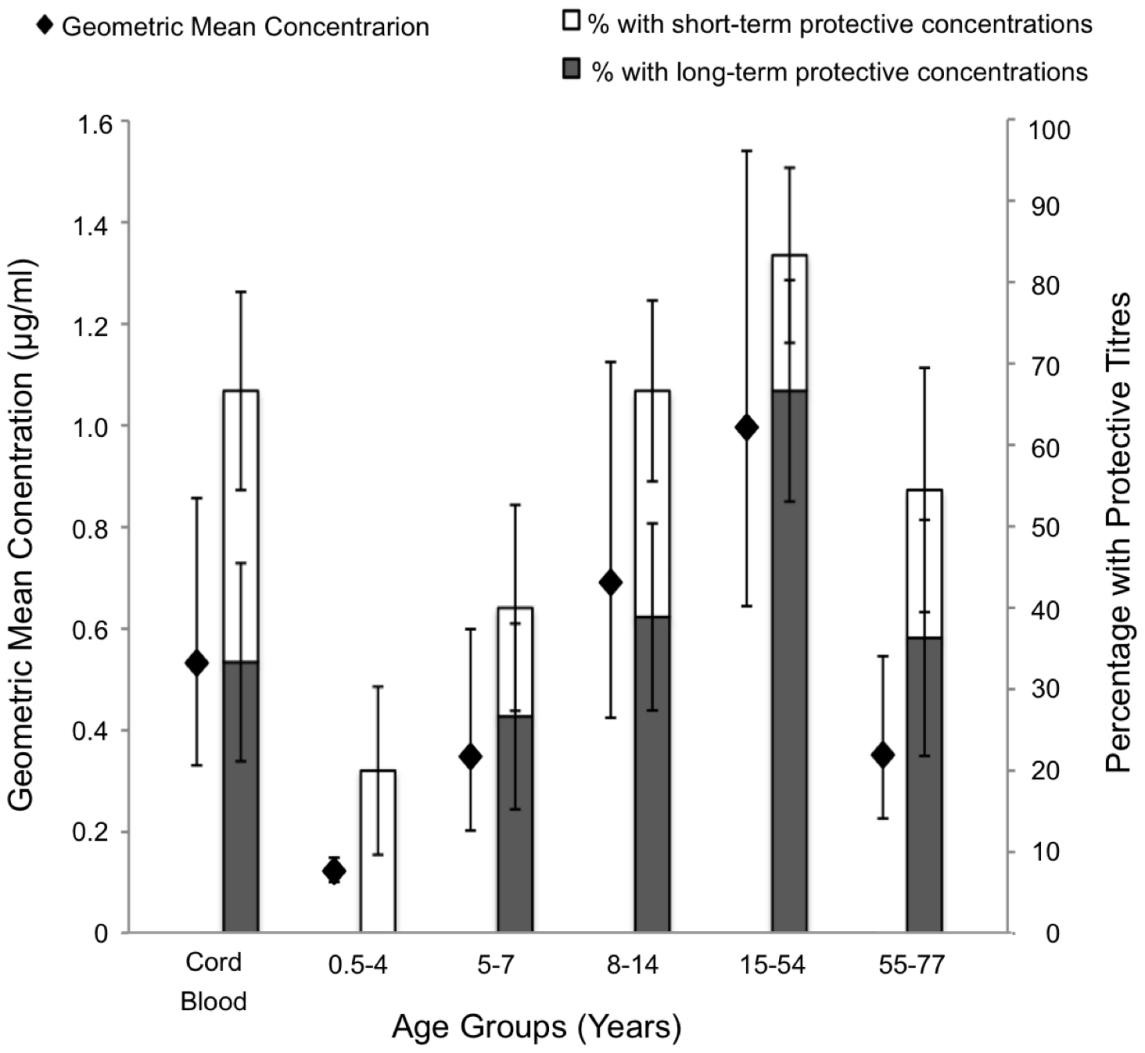
Mean Anti-Hib Antibody Concentrations and Percentage of Participants with Protective Levels. Only 20% of children under 5 years old have protective (>0.15 µg/ml) antibody levels, rising to 83% of 15–54 year-olds. Geometric mean anti-polyribosylribitol phosphate IgG concentrations for each age group are plotted on the left y-axis (±SE). The percentage of participants with antibody concentrations >0.15 µg/ml (‘short-term protection’: the height of the entire column) and >1 µg/ml (‘long-term’ protection: the height of only the shaded column) are plotted on the right y-axis (±SE). Sample sizes (n): Cord Blood (n = 15); 0.5–4 yrs (n = 15); 5–7 yrs (n = 15); 8–14 yrs (n = 18); 15–54 yrs (n = 12); 55–77 yrs (n = 11).

Seventy-one samples from participants aged 7 months to 77 years and 15 cord blood samples were analysed for anti-Hib (anti-PRP) IgG. Participants were grouped by age into 6 groups: cord blood (n = 15), 0.5–4 yrs (n = 15); 5–7 yrs (n = 15); 8–14 yrs (n = 18); 15–54 yrs (n = 12); 55–77 yrs (n = 11). For each age group the geometric mean antibody concentration (GMC) was calculated together with the percentage of participants with concentrations above the accepted correlates of ‘short-term’ (>0.15 µg/ml) and ‘long-term’ (>1 µg/ml) protection (Fig. 1).

In the 0.5–4 year-olds the anti-PRP GMC and percentage protected were low in relation to the pre-defined protective thresholds; of note, no children in this age-group had an anti-PRP concentration >1 µg/ml. The GMC and percentage with antibody concentrations greater than the protective thresholds were higher in 5–7 year-olds, with the majority of this age-group (60%) having antibody levels >0.15 µg/ml. GMCs and percentages with protective concentrations were higher still in 8–14 year-olds, and highest in the 15–54 year-olds. In children up to 12 years-old 60% had anti-PRP antibody concentrations below the threshold of detection in contrast to none of those participants between the ages of 13 and 49 (data not shown). The GMC and percentages with protective antibody concentrations were lower in the over 55 age-group than in the 15–54 year-olds.

## Discussion

To our knowledge, this is the first Hib seroepidemiological study from Nepal. The general pattern of initially high anti-PRP antibody concentrations from transplacentally transferred maternal antibody, waning in early infancy, subsequent seroconversion throughout childhood, and waning levels in the elderly, is consistent with Hib seroepidemiological data reported in other settings [15]. Previous studies have demonstrated the relationship between anti-PRP antibodies and protection from invasive disease [15]. It is presumed that anti-PRP antibody is acquired through nasopharyngeal carriage of Hib or exposure to organisms possessing antigens that are immunologically cross-reactive with PRP. For any age cohort, the anti-PRP antibody concentrations give an indication of the proportion of individuals susceptible to Hib infection whilst the rate of acquisition of anti-PRP antibody between age cohorts should reflect the degree of exposure to Hib or cross-reactive antigens. In our study, 80% of children under 5 were serologically susceptible to invasive Hib disease with concentrations of anti-PRP antibody below the threshold of protection (<0.15 µg/ml). Even in late childhood, a substantial proportion of individuals still did not have protective concentrations of anti-PRP antibody (Fig. 1).

### Anti-PRP Acquisition

Comparisons of antibody levels between different Hib seroepidemiological studies are limited by significant inter-laboratory variability in the anti-PRP assay, and even between different labs using the same anti-PRP ELISA [16]. It is therefore difficult to use age-specific serology data to infer disease incidence. However, the profile of anti-Hib antibody acquisition throughout childhood can be compared and clearly differs between countries (see Supporting Information, Figure S1 & Table S1, in File S1). Some studies find that a low proportion of young children have protective concentrations (>0.15 µg/ml) but a rapid increase in the proportion of older children who are protected. For example, in Burkina Faso whilst only 9% of children under 5 years of age had concentrations >0.15 µg/ml, 75% of those aged 4–14 years were protected [17]. This suggests a relatively high degree of exposure to Hib in the under 5 s, and correlates with Hib meningitis incidence data from Burkina Faso [18], and other African countries, which suggest a high proportion of cases in infants, and a high incidence in the under 5 s. Carriage data from West Africa also support a high exposure to Hib in this region [19]. Seroepidemiological studies from some other regions, including the UK [20], display a more gradual increase in the proportion protected with increasing age. Overall, however, it is difficult to identify a clear relationship between the different population serologic profiles (Figure S1 & Table S1, in File S1) and either the estimated incidence of Hib meningitis under 5, or the proportion of Hib meningitis cases in infants [18]. Contributing factors that may obscure a relationship between anti-PRP antibody and Hib exposure are the variability in anti-PRP assays and the levels of exposure to cross-reactive antigens [21]. Of all studies identified, our data demonstrated one of the slowest rates of acquisition of protection (<0.15 µg/ml), from 20% under 5 years of age, to 40% in those aged 5–7 years, 67% in 8–14 year-olds, and 83% at 15–54 years. This suggests a relatively low level of Hib exposure compared with that observed in other countries in the pre-vaccine era.

### Carriage

In populations with a known burden of invasive Hib disease, reported carriage rates vary from 3 to 9% in children under 5 years of age [22]. Such data for Nepal are sparse, but Williams *et al.* reported a Hib carriage prevalence of 5% in children aged 3 months to 12 years in the pre-vaccination era [4]. This is comparable to countries such as the USA and UK [23], [24], where disease incidence has been sufficient to warrant routine immunization.

### Disease incidence

Although there have been no population incidence data for invasive Hib disease reported from Nepal, two studies prior to the introduction of routine Hib immunisation from Kathmandu demonstrated that Hib was the second most common cause of meningitis in children after *Streptococcus pneumoniae* [25], [26]. Hib was identified in 25% of cases (n = 77) of bacterial meningitis (with established aetiology) in children under 5 [26], which is low compared with most studies globally, as meta-analysed by WHO [18], but still indicative of a significant burden of disease. By inference, Hib pneumonia is likely to be prevalent but difficult to demonstrate as cases are rarely bacteraemic [25], [26]. Five South-East Asian studies in the WHO analysis [18], and one of the studies from Kathmandu [26], demonstrated a high proportion of total Hib meningitis cases occurring in infancy. Although it has been suggested that the proportion of infant cases is positively related to overall disease incidence, the correlation is relatively weak, making inferences about incidence unreliable for any individual country.

### Limitations

The limitations of our study include the small sample size which precludes a more detailed age-specific analysis of Hib immunity acquisition in childhood, for example in those under 2 years who would be expected to have impaired anti-PRP responses to natural Hib exposure. There were only 5 children under 2, none of whom had protective levels of antibody (>0.15 µg/ml). Also, the small blood volumes in young children were insufficient to allow comparisons of antibody avidity. Avidity has previously been proposed as a biomarker for successful induction of immunological memory, with increased avidity representing more functional antibody [9]. Measuring antibody avidity may identify an effective immunological memory (secondary to ‘natural priming’) in subjects with low antibody concentrations [27]. Selection bias may have been introduced by the hospital-based nature of this study, the patient charges at Patan Hospital (although local medical staff consider that the costs are not sufficient to deter attendance) [25], and the attributes of patients willing to participate in a research study of this kind. Finally the waning anti-PRP levels measured in the elderly may reflect comorbidities, which were not solicited in this study.

### Implications

The importance of Hib in Asia has previously been questioned. However, our data suggest that at least 83% of the Kathmandu population had been naturally exposed to Hib, or cross-reactive antigens, before adulthood. Although our seroepidemiological data are most compatible with a lower level of exposure to Hib carriage, previous data on Hib carriage prevalence [4], and meningitis aetiology [25], [26] still indicate the importance of Hib disease in Nepali children. Furthermore, we identified a relatively prolonged period for which children are at risk of a disease for which carriage and aetiological studies support a significant burden of invasive illness, with the majority of children under 8 being susceptible to Hib. Now that a Hib conjugate vaccine is included in the national immunization schedule, we would expect Hib transmission to be low, through reduced acquisition amongst vaccinees. Since this will in turn reduce the opportunity for natural boosting of Hib antibody levels, ongoing serological surveillance could be used to inform the need for booster doses of vaccine, using data in this study as a baseline.

## Supporting Information

File S1 **Table S1 and Figure S1.** Comparison of Hib seroprevalance studies in the pre-vaccine era.
(DOC)
