# Supplementary material for: The Seroepidemiology of Haemophilus influenzae Type B Prior to Introduction of an Immunization Programme in Kathmandu, Nepal
Source: PLoS One. 2014 Jan 22;9(1):e85055. doi: 10.1371/journal.pone.0085055 (PMC3898912; doi:10.1371/journal.pone.0085055)
Supplement: File S1 — Table S1 and Figure S1. Comparison of Hib seroprevalance studies in the pre-vaccine era. (DOC) [file pone.0085055.s001.doc]

**File S1 - SUPPORTING INFORMATION**

**TABLE S1.** Comparison of Hib seroprevalance studies in the pre-vaccine era

| **Age-group in months / years** | **Study country**  **(sample size)** | **% with protective titres >0.15 μg/ml** | **Reference** |
| --- | --- | --- | --- |
|  |  |  |  |
| Cord blood | Australian Aboriginals (30) | 43 |  |
| Cord blood | Australian Europeans (53) | 62 |  |
| Cord blood | UAE (48) | 65 |  |
| Cord blood | Nepal (15) | 67 | *This study* |
| Cord blood | Gambia (86) | 67 |  |
| 0 mo | China (56) | 88 |  |
| Cord blood | USA (47) | 89 |  |
| Cord blood | Burkina-Faso (89) | 100 |  |
|  |  |  |  |
| 6-47 mo | Burkina-Faso (105) | 9 |  |
| 6-59 mo | Nepal (15) | 20 | *This study* |
| 6-47 mo | France (113) | 23 |  |
| 6-47 mo | Singapore (*) | ~28 |  |
| 5-47 mo | Italy (147) | 33 |  |
| 18-24 mo | India (123) | 35 |  |
| 0-59 mo | Turkey (395) | 42 |  |
| 4-47 mo | Japan (60) | 45 |  |
| 17-19 mo | USA (313) | 46 |  |
| 6-47 mo | Finland (558) | 49 |  |
| 12-60 mo | India (73) | 56 |  |
| 6-23 mo | China (173) | 57 |  |
| 12-59 mo | UK (*) | ~57 |  |
| 6-60 mo | Turkey (242) | 65 |  |
| 13-60 mo | Iran (312) | 89 |  |
|  |  |  |  |
| 5-7 yrs | Nepal (15) | 40 | *This study* |
| 5-10 yrs | India (130) | 46 |  |
| 4-6 yrs | Japan (20) | 60 |  |
| 2-9 yrs | China (71) | 61 |  |
| 5-9 yrs | Turkey (365) | 65 |  |
| 4-14 yrs | France (26) | 69 |  |
| 5-7 yrs | UK (*) | ~74 |  |
| 4-5 yrs | Singapore (*) | ~75 |  |
| 4-14 yrs | Burkina-Faso (20) | 75 |  |
| 4-6 yrs | Finland (273) | 79 |  |
| 4-6 yrs | Italy (88) | 80 |  |
| “By 6 yrs” | Hong Kong Chinese (*) | 90 |  |
| 4-5 yrs | Cuba (974) | 100 |  |
|  |  |  |  |
| 8-14 yrs | Nepal (18) | 67 | *This study* |
| 10-19 yrs | Turkey (160) | 72 |  |
| 10-19 yrs | China (86) | 80 |  |
| 7-12 yrs | Japan (20) | 85 |  |
| 8-15 yrs | UK (*) | ~89 |  |
| 8-16 yrs | Singapore (*) | ~93 |  |
| 7-16 yrs | Italy (82) | 93 |  |
|  |  |  |  |
| 20-49 yrs | Turkey (546) | 82 |  |
| 15-55 yrs | Nepal (12) | 83 | *This study* |
| 20-49 yrs | China (330) | 86 |  |
| 17-70 yrs | Italy (100) | 95 |  |
| 18-23 yrs | Japan (107) | 96 |  |
| 17-21 yrs | Singapore (*) | ~97 |  |
| “Adults” | Finland (49) | 100 |  |
|  |  |  |  |
| 55-77 yrs | Nepal (11) | 55 | *This study* |
| 50-82 yrs | Turkey (247) | 79 |  |
| 50-60 yrs | China (79) | 96 |  |
|  |  |  |  |

Data were extracted from Medline English-language references, and reorganised where necessary to match, as closely as possible, the age-groups used in our study. Some references were excluded, including those dealing solely with infants (including control samples from vaccine immunogenicity studies), single-sex studies (e.g. of pregnant women), where another study with similar data had already been included from the same country and age-group, or where comparable data could not be extracted or accessed.

Two studies also reported data for median anti-PRP concentration or anti-PRP GMC, which were comparable with our own. Similarly, the profile of the development of anti-Hib immunity with age was slower in our data from Kathmandu in comparison with these studies from the UK and Italy (data not shown).

* Although full numerical data were not published/accessible for these three references, they did represent countries not otherwise included, and percentages protected were given or could be estimated from graphical data.


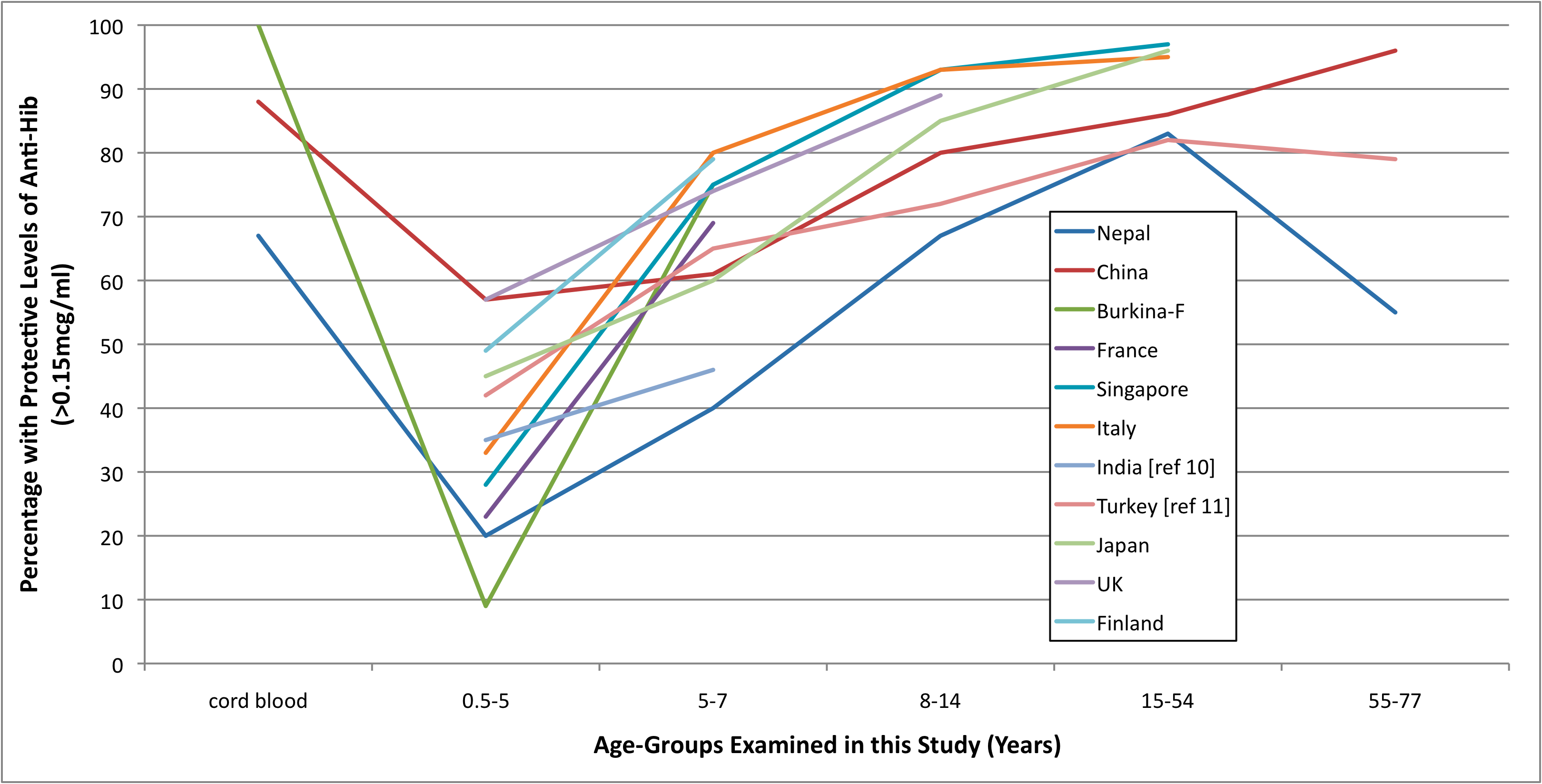
**Figure S1 – Anti-Hib Population Serological Profiles**

Data from Table S1 shows the rate of acquisition of anti-Hib immunity across different age groups, in different populations. Studies with 2 or more data points in the 0.5-77 years age-groups are included, with data from 1 reference per country for clarity. Age-groups from our study are indicated on the x-axis but age-groups in the other studies are as described in Table S1. There is a range of rates of acquisition of Hib immunity, from countries with gradual acquisition throughout childhood, such as Nepal, to those with rapid acquisition in pre-school children, such as Burkina Faso.

**References for Table & Figure S1**

1. Guthridge S, McIntyre P, Isaacs D, Hanlon M, Patel M (2000) Differing serologic responses to an haemophilus influenzae type b polysaccharide-neisseria meningitidis outer membrane protein conjugate (PRP-OMPC) vaccine in australian aboriginal and caucasian infants - implications for disease epidemiology. Vaccine 18: 2584-2591.

2. Uduman SA, Bener A, Koshy JK, Hussen SM (1995) Natural immunity to Haemophilus influenzae type b in healthy infants on Al Ain, UAE. European Journal of Public Health 5: 291-292.

3. Mulholland K, Suara RO, Siber G, Roberton D, Jaffar S, et al. (1996) Maternal immunization with Haemophilus influenzae type b polysaccharide-tetanus protein conjugate vaccine in The Gambia. JAMA : the journal of the American Medical Association 275: 1182-1188.

4. Fu C, Xu J, Liang J (2009) Seroprotection to Haemophilus influenzae type b infection in a healthy population of Guangzhou, China. The Pediatric infectious disease journal 28: 757.

5. Englund JA, Glezen WP, Thompson C, Anwaruddin R, Turner CS, et al. (1997) Haemophilus influenzae type b-specific antibody in infants after maternal immunization. The Pediatric infectious disease journal 16: 1122-1130.

6. Tall F, Elola A, Vincent-Ballereau F, Prazuck T (1994) [Anti-Haemophilus influenzae b (Hib) natural immunity in children in Burkina Faso]. Archives de pediatrie : organe officiel de la Societe francaise de pediatrie 1: 143-146.

7. Ballereau F, Speich M, Apaire-Marchais V (1999) Natural Haemophilus influenzae type b capsular polysaccharide antibodies in 412 infants and children from West Africa (Burkina-Faso) and France: a cross-sectional serosurvey. European journal of epidemiology 15: 577-582.

8. Lolekha S, Cooksley G, Chan V, Isahak I, Ismael S, et al. (2000) A review of Hib epidemiology in Asia. The Southeast Asian journal of tropical medicine and public health 31: 650-657.

9. Sansoni A, Rappuoli R, Viti S, Costantino P, Fanti O, et al. (1992) Immunity to Haemophilus influenzae type b on sample population from central Italy. Vaccine 10: 627-630.

10. Acharya D, Desai A, Nanavaty N, Pandit A, Patel V, et al. (1995) Evaluation of immunogenicity and tolerance of single dose haemophilus influenzae type B (PRP-T) vaccine. Indian pediatrics 32: 1077-1082.

11. Sonmez C, Coplu N, Kurtoglu D, Esen B, Crowley Luke A, et al. (2010) Levels of Haemophilus influenzae type B (Hib) antibody in Turkey before routine immunization. Turkish Journal of Medical Sciences 40: 959-964.

12. Ishiwada N, Fukasawa C, Inami Y, Hishiki H, Takeda N, et al. (2007) Quantitative measurements of Hemophilus influenzae type b capsular polysaccharide antibodies in Japanese children. Pediatrics international : official journal of the Japan Pediatric Society 49: 864-868.

13. Holmes SJ, Murphy TV, Anderson RS, Kaplan SL, Rothstein EP, et al. (1991) Immunogenicity of four Haemophilus influenzae type b conjugate vaccines in 17- to 19-month-old children. The Journal of pediatrics 118: 364-371.

14. Peltola H, Kayhty H, Sivonen A, Makela H (1977) Haemophilus influenzae type b capsular polysaccharide vaccine in children: a double-blind field study of 100,000 vaccinees 3 months to 5 years of age in Finland. Pediatrics 60: 730-737.

15. Acharya D, Bhave S, Joshi V, Bavdekar A, Pandit A (1997) Haemophilus influenzae type b vaccine in India: need and timing, immunogenecity and tolerance. Indian pediatrics 34: 9-15.

16. Trotter CL, McVernon J, Andrews NJ, Burrage M, Ramsay ME (2003) Antibody to Haemophilus influenzae type b after routine and catch-up vaccination. Lancet 361: 1523-1524.

17. Ocaktan E, Ozyurda F, Akar N (2004) Natural immunity to Haemophilus influenzae type B in children of Ankara, Turkey. Pediatrics international : official journal of the Japan Pediatric Society 46: 280-284.

18. Jahromi AS, Rahmanian K (2012) Natural immunity to Haemophilus influenzae Type b in children, South of Iran: need for vaccination. Pakistan Journal of Biological Sciences 15: 160-163.

19. Lau YL, Yung R, Low L, Sung R, Leung CW, et al. (1998) Haemophilus influenzae type b infections in Hong Kong. The Pediatric infectious disease journal 17: S165-169.

20. Torano Peraza G, Hernandez Vadell I, Toledo Romani ME, Baly Gil A, Tamargo Martinez I, et al. (2004) Naturally acquired immunity to Haemophilus influenzae type B in healthy Cuban children. Memorias do Instituto Oswaldo Cruz 99: 687-689.
